# A Contribution to the Bacteriological Study of B. Influenzae

**Published:** 1918-11

**Authors:** 


**A Contribution to the Bacteriological Study of B. Influenzae.** By MM. Barbie and Leclerc. *La Presse Médicale*, October 3, 1918.

The present epidemic of influenza is comparable to the great epidemic of 1889. The disease presents the characteristic features that were noted in 1889 : rapid spread, sudden onset, very marked infectivity.

This epidemic, although rather mild during the months of May and June, gave rise to very severe and complicated cases. A particular feature of the present epidemic is the incidence of severe complications, such as pneumonia and bronchopneumonia, even during the summer.

A certain number of micro-organisms have been supposed to be the etiologic agents of influenza : the pneumococcus and streptococcus seem responsible for a certain number of cases, but their specificity has never been demonstrated.

In 1892, Pfeiffer was convinced that the bacillus named after him was the only infective agent for influenza. This bacillus is present, however, in many other diseases. Its presence has been ascertained in the sputum of tuberculous patients; it seems responsible also for certain cases of meningitis, and for the pulmonary complications arising in childhood after " rash-fevers ".

*Slight Cases of Influenza.* The authors have made systematic bacteriological researches in order to discover the specific agent of that disease.

All blood cultures tried in glucosated broth remained sterile.

Never did they detect Pfeiffer's bacillus on examining smears of blood and mucus.

Pneumococci were found in the sputum of some patients, but never in very large numbers.

Guinea-pigs injected intraperitoneally with 1 1/2 or 2 cc. of blood, removed aseptically from the patients during a pyretic attack of the disease, suffered for a few days from periodical rises of temperature of 2 or 3° in the morning and evening.

No micro-organisms could be detected in the blood of the guinea pigs thus injected.

In mild cases of influenza, neither Pfeiffer's bacillus, nor any of the bacilli generally responsible for the lung diseases, could be detected.

*Influenza with Severe Complications.* On the other hand, a bacillus possessing most of the characteristic features of Pfeiffer's bacillus could be isolated in a certain number of cases where pneumonia, bronchopneumonia, or pleurisy had developed as complications to influenza.

1) In 7 out of 19 blood cultures, a motionless, gram-negative, strictly aerobic bacillus was discovered. This organism developed abundant strains on blood-agar. It was grown with difficulty on glucose agar, and did not grow at all on ordinary laboratory media.

This bacillus is only very slightly visible when examined between a glass slide and cover-slip; it is best visible after intense dyeing with diluted Ziehl's fuchsin. It has a bacillar or coccobacillar shape.

In another series of blood cultures, 5 results out of 10 were positive for this Pfeiffer bacillus-like organism.

2) An organism of the same kind was found together with pneumococci and streptococci in 10 cultures made from the fluids collected in the pleural cavity in cases of purulent pleurisy. Guinea-pigs injected with 1 cc. of these pleural fluids died 12 hours after the injection. Post-mortem examination of these animals showed that the peritoneal exudate contained Pfeiffer's bacilli in very large numbers.

3) In most cases Pfeiffer's bacillus was found to be associated with other organisms, diplococci principally. Some of these had all the characteristic features of pneumococci. Others were of the streptococcus kind, and even of the streptococcus mucosus variety.

4) Never was the pneumococcus the predominant germ in the sputum. In some cases a pure growth of Friedlander's pneumobacillus was obtained from the sputum. Never was Pfeiffer's bacillus detected there.

Finally Pfeiffer's bacillus was discovered, together with pneumococci, streptococci, and pneumobacilli, in the blood and pleural exudate of patients suffering from influenza, especially in severe cases, and during the last days of the disease. But as yet it has not been ascertained whether or not this bacillus may be considered as the etiologic agent of influenza.

